# A Markov computer simulation model of the economics of neuromuscular blockade in patients with acute respiratory distress syndrome

**DOI:** 10.1186/1472-6947-6-15

**Published:** 2006-03-15

**Authors:** Alex Macario, John L Chow, Franklin Dexter

**Affiliations:** 1 Department of Anesthesia, Stanford University School of Medicine, Stanford, CA 94305, USA; 2 Health Research & Policy, Stanford University School of Medicine, Stanford, CA 94305, USA; 3 Division of Management Consulting, Department of Anesthesia, University of Iowa, Iowa City, Iowa, 52242, USA

## Abstract

**Background:**

Management of acute respiratory distress syndrome (ARDS) in the intensive care unit (ICU) is clinically challenging and costly. Neuromuscular blocking agents may facilitate mechanical ventilation and improve oxygenation, but may result in prolonged recovery of neuromuscular function and acute quadriplegic myopathy syndrome (AQMS). The goal of this study was to address a hypothetical question via computer modeling: Would a reduction in intubation time of 6 hours and/or a reduction in the incidence of AQMS from 25% to 21%, provide enough benefit to justify a drug with an additional expenditure of $267 (the difference in acquisition cost between a generic and brand name neuromuscular blocker)?

**Methods:**

The base case was a 55 year-old man in the ICU with ARDS who receives neuromuscular blockade for 3.5 days. A Markov model was designed with hypothetical patients in 1 of 6 mutually exclusive health states: ICU-intubated, ICU-extubated, hospital ward, long-term care, home, or death, over a period of 6 months. The net monetary benefit was computed.

**Results:**

Our computer simulation modeling predicted the mean cost for ARDS patients receiving standard care for 6 months to be $62,238 (5% – 95% percentiles $42,259 – $83,766), with an overall 6-month mortality of 39%. Assuming a ceiling ratio of $35,000, even if a drug (that cost $267 more) hypothetically reduced AQMS from 25% to 21% and decreased intubation time by 6 hours, the net monetary benefit would only equal $137.

**Conclusion:**

ARDS patients receiving a neuromuscular blocker have a high mortality, and unpredictable outcome, which results in large variability in costs per case. If a patient dies, there is no benefit to any drug that reduces ventilation time or AQMS incidence. A prospective, randomized pharmacoeconomic study of neuromuscular blockers in the ICU to asses AQMS or intubation times is impractical because of the highly variable clinical course of patients with ARDS.

## Background

Management of patients with acute respiratory distress syndrome (ARDS) in the intensive care unit (ICU) is clinically challenging and costly [1]. In ARDS patients with refractory hypoxemia, neuromuscular blocking agents may facilitate mechanical ventilation and improve oxygenation. However, prolonged recovery of neuromuscular function and development of acute quadriplegic myopathy syndrome (AQMS) can occur [2]. While a variety of neuromuscular blockers have been utilized, it remains unclear which agent provides the optimal clinical benefit relative to the drug acquisition cost [3]. For example, using average wholesale prices, the cost of treating an ARDS patient with cisatracurium for 3.5 days is approximately $267 more than if the patient received vecuronium. (Table 1 and 2)

**Table 1:** Drug acquisition costs for 3.5 day cycle of neuromuscular blockade using average wholesale price (AWP)

	Load (mg)	Mg infusion rate/hr	AWP vial ($)	Vial size (mg)	Cost/mg ($)	Load cost ($)	Cycle infusion cost ($)	Daily cost ($)	**Total cost per cycle ($)**	Total mgs	Opened vials	Total cost per cycle with open vials ($)
Vecuronium	7	4	9.69	10	0.97	6.78	326	93	**332**	343	35	339
Cisatracurium	14	8	174.5	200	0.87	12.22	586	168	**599**	686	4	698
Cost difference =									**$ 267**			$ 359

**Table 2:** Drug acquisition costs for 3.5 day cycle of neuromuscular blockade using average selling price

	Load (mg)	Mg infusion rate/hr	Mean sales price ($)	Vial size (mg)	Cost/mg ($)	Load cost ($)	Cycle infusion cost ($)	Daily cost ($)	**Total cost per cycle ($)**	Total mgs	Opened vials	Total cost per cycle with open vials ($)
Vecuronium	7	4	3.58	10	0.36	2.5	120	34	**123**	343	35	125
Cisatracurium	14	8	119.5	200	0.60	8.4	402	115	**410**	686	4	478
Cost difference =									**$ 287**			$ 353

Assessing the clinical and economic consequences of pharmacological interventions in ARDS patients is difficult. The reasons include a heterogeneous patient population, a wide range of supportive interventions, and complex causes of the patient's condition. For example, to conduct a clinical trial with sufficient statistical power to detect a 10% absolute decrease in the incidence of AQMS, a randomized clinical trial would have to enroll 800 patients in each of the two groups, assuming censoring due to mortality is 30%. In such situations where clinical studies are expensive and complicated to complete, computer modeling is an appropriate initial approach to yield insights.

The goal of this study was to address a hypothetical question via computer modeling: Would a reduction in intubation time of 6 hours and/or a reduction in the incidence AQMS from 25% to 21% in ARDS patients, provide enough benefit to justify an additional expenditure of $267 [2,4]?

## Methods

### Overview of computer model

The numerator in the incremental cost-effectiveness ratio takes into consideration the additional costs that one intervention imposes over another. The denominator considers the incremental improvement in health related quality of life calculated as quality-adjusted life-years (QALY). Both costs and QALYs need to be considered together, otherwise death becomes the least costly option.

Using the results of our literature review, we simulated the rates of healing and complications associated with patients with ARDS, computed associated incremental costs, assumed a societal perspective for the analysis as recommended by an expert panel, estimated quality of life for relevant health states, and performed a sensitivity analysis to evaluate the impact of changing key variables [5].

#### Markov model

Conventional models based on decision trees are limited in their ability to describe events that can occur multiple times in the care of a patient (e.g., ICU readmissions). A Markov model is a mathematical representation of patients in a series of health states. Such a model provides a tool to deal with multiple clinical uncertainties because the study can be repeated in successive iterations by varying parameters to address "What if?" questions. The use of Markov models is particular relevant in the ICU settings given the constantly changing nature of the patients' disease conditions and treatments. This methodology has been applied successfully in studying the incidence of nosocomial infections in critically ill patients, and the mortality of ICU patients with sepsis [6,7].

#### Base case

**Figure 1:**
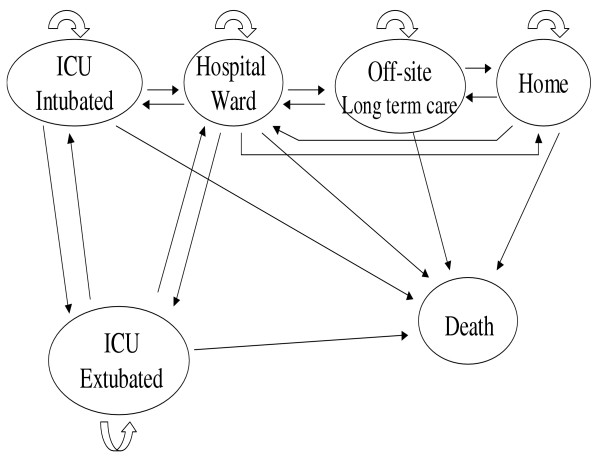
**Markov model for patient with ARDS receiving neuromuscular blockade**. Simulated patients were classified into 6 health states. Patient progression was divided into 3.5-day cycles over a 6-month period.

For our study, we chose the following base case; a 55 year-old man is admitted to the ICU because of ARDS secondary to pneumonia (pneumonia sepsis is the most common etiology for ARDS and receives neuromuscular blockade for 3.5 days. These hypothetical patients were modeled to be in 1 of 6 mutually exclusive health states: ICU-intubated, ICU-extubated, hospital ward, long-term care, home, or death, over a period of 6 months. (Figure 1)

A 3.5-day cycle time was chosen because it approximates the time of important clinical changes. Six months was chosen because it allows the model to consider the natural progression and resolution of the disease over a reasonable time frame. We also modeled a 1-month period. Choosing a longer period such as 12 or 24 months period would have been unnecessarily long.

#### Net monetary benefit

The incremental cost-effectiveness ratio has poor statistical properties within the range of values relevant to this study [8]. If two drugs provide similar effectiveness, then trying to calculate the incremental cost-effectiveness ratio results in division by zero. Difficulties arise in how to place a monetary value on a clinical improvement. This valuation is performed by assigning a monetary value to a unit of effectiveness (Z), and multiplying it by the net number of units of effectiveness achieved. The value Z represents the maximum amount that society would be willing to pay for the incremental improvement in outcome (and therefore its maximum value). Medical interventions with a cost-effectiveness of less than $35,000 (Z) per QALY are generally considered to represent acceptable value for money, i.e., be cost-effective. As there is no correct or well-accepted value of Z for a given clinical improvement, we tested a range of Z values from 0 to $100,000. The net monetary benefit addresses these concerns by assigning a monetary value to the incremental benefit achieved, and subtracting from this the incremental cost of achieving this benefit [9].

A positive net monetary benefit implies that the cost of a new therapy is less than the value of the additional benefit achieved. A negative net monetary benefit implies that an intervention should be rejected, as its costs are higher than the value of the benefit achieved [10].

#### Hospital costs and health related quality of life

Total hospital costs can be separated into fixed (which do not change in proportion to the number of ICU cases) and variable components. For example, from the facility's perspective, nursing care time may be considered a fixed cost, as staff is paid regardless of whether there is one more or one less ICU patient. However, we assumed that having a provider take care of an ARDS patient is an incremental cost to society, as is commonly done in cost-effectiveness studies, to reflect that, from society's point of view, there is a cost for the provider's time and expertise.

Using data gathered from the literature and utilizing a "bottom up" cost methodology, we estimated direct medical costs per day as the hypothetical patients progressed thru the various health states. We assumed that 10% of the costs assigned to any of the health states are for physicians' professional services [11]. We assumed that daily costs in any of the health states are linear, meaning that the first ICU day, for example, is equally costly as subsequent ICU days.

Quality-adjusted life-years include a length of time component (e.g., one year) and a quality of life component (i.e., utility). Health utility is the numerical valuation of one's health-related quality of life on a linear scale from 0.00 (death) to 1.00 (perfect health). For example, one quality-adjusted life-year for an individual in perfect health (with a utility = 1.0) for one year (QALY = 1) is considered equivalent to two years in a health state with utility = 0.5. (QALY = 1). The advantage of using QALYs is that they combine number of years saved as well as the quality of life of those years.

Directly ascertaining utilities in critically ill patients is done infrequently and is methodologically difficult [12]. Published utilities from subgroups of ICU patients have been confirmed with the EuroQol scale and the Rosser index [13-15]. Patients in the EuroQol^© ^EQ-5D scale are classified into one of 243 (3^5^) health states (mobility, self-care, usual activity, pain, mood) [16]. Each state is scored from 1 (normal) to 3 (the most impaired). For example, a mobility score of "1" indicates "no problems in walking about," while a "3" is "confined to bed." The scores for the five states can be assigned a utility valuation from the general public. For example, a EuroQol mobility (3), self-care (3), usual activity (3), pain (2), mood (1) signifies a utility of 0.08. In contrast, EuroQol mobility (1), self-care (1), usual activity (2), pain (1), mood (2) signifies a utility of 0.65.

In the Rosser classification of illness, assigning levels of disability and distress to each health state determine the quality of life of a patient. For example, Rosser Disability level VII with Distress level B (mild) indicates a utility of 0.8.

Since health values of seriously ill patients vary widely, we incorporated a wide range of quality of life for each health state, assuming no state was worse than death. (Table 3)

**Table 3:** Costs and utilities used for each health state for computer modeling

Health state	Cost per day ($)	Range		Utility	Range
		Low ($)	High ($)		
ICU intubated	2200	1400^24,25^	3700^26,27^	0.1	0.08–0.15
ICU extubated	1500^28^	700^29^	2400	0.2	0.1 – 0.3
Hospital ward	700	450^30,31^	1700	0.5	0.25–0.56^32^
Long term care	350	100^33^	925	0.65	0.45^34 ^– 0.77^35,36^
Home	0			0.80	0.78^37 ^– 0.92^38,39^

### Question asked of computer modeling

The computer modeling of the natural history of ARDS used incidences of progressing through the health states as estimated from the articles retrieved from the literature. We then asked the question, "Would a reduction in intubation time of 6 hours and/or a reduction in the incidence AQMS from 25% to 21% in ARDS patients, provide enough benefit to justify an additional expenditure of $267? Four scenarios were specifically considered (1) the agent reduces the incidence of myopathy from 25 to 21%, (2) the agent reduces the duration of mechanical ventilation by 6 hours, (3) both; (4) neither.

### Sensitivity analyses

The probabilistic sensitivity analysis considered uncertainties in all probabilities, utilities, and costs simultaneously. Mean values for the net monetary benefit were calculated for results of N = 10,000 Monte-Carlo simulations (@Risk 4.0, Newfield, NY, Palisade Corporation). Triangular distributions were used for parameter values, with the mode being the base case and the 5th and 95th percentiles being the lower and upper limits of the ranges reported [17].

All costs are reported in year 2004 U.S. dollars. We discounted all future costs and quality-adjusted life-years at 3% per annum [18].

## Results

ARDS patients receiving a neuromuscular blocker have a high mortality, and unpredictable outcome, which results in large variability in costs per case. If a patient dies, there is no benefit to a drug that reduces ventilation time or AQMS incidence.

### Computer modeling

The estimated mean total cost for an ARDS patient receiving standard care for 6 months was $62,238 (5% – 95% percentiles $42,259 – $83,766; median, $61,885). Our computer model predicted that out of 100 hypothetical patients with ARDS, 39% would be expected to be dead after 6 months. (Table 4)

**Table 4:** Fraction of patients in each health state after 1 month and 6 months

Health state	After 1 month	After 6 months
ICU intubated	28%	6%
ICU extubated	23%	6%
Hospital ward	9%	4%
Off site long term care	9%	18%
Home	8%	28%
Dead	23%	39%

Results of literature review are in Tables 5, 6, 7.

**Table 5:** Randomized control trials of treatments for ARDS published after 1998

**Author**	**Derdak**^40^	**Eisner**^41^	**Gattinoni**^42^	**ARDS network**^43^	**Ely**^44^	**Lagneau**^45^
Subjects	All comers ARDS	Pneumonia ARDS	All comers ARDS	All comers ARDS	ARDS network (age <70)	Pa02/FiO2 < 200
Intervention	Ventilation-controlled or high frequency	Tidal volume 12 ml/kg or 6 ml/kg	Supine vs. prone	Placebo vs. ketoconazole	Tidal volume 12 ml/kg or 6 ml/kg	Cisatracurium 0/4 twitches or 2/4 twitches
# of subjects	147	320	304	234	729	102
Mean age (yrs)	49	51	58	53	46	56
Severity of illness	22**	84***	40*	81***	73***	41*
Pa02/FiO2	113	133	127	145		130
ICU days					19	
Days on ventilator	21				10	
In-hospital mortality				35%		45%
% with unassisted breathing at 1 mth		57%		59%		
10 day mortality			25%, 21%			
30 day mortality	52%, 37%				25%	
6 mth mortality	59%, 47%	36%	59%, 63%		30%	
Ventilator free days in first 28 days				10		
reintubation					7.5%	

**Table 6:** Cohort studies of patients with ARDS published after 1999

**Author**	**Estenssoro**^46^	**Luhr**^47^	**Davidson**^48^	**Arroliga**^49^	**Reynolds**^50^	**Angus**^13^	**Fialkow**^51^
Study type	prospective	prospective	prospective	retrospective	retrospective	prospective	retrospective
# of subjects	235	221	127	66	720	200	30
Mean age (yrs)	55	61	39	60		49	51
Severity of illness	21**	19**	73***	23**		17**	18**
Pa02/FiO2	141	131		111			
ICU days (range)				12		16 (0–93)	21
Ventilator days							
Hospital days (range)			28(1–150)			26 (0–117)	44
3 day mortality	34%						
ICU mortality				47%			47%
In-hospital mortality	58%		43%		36%		
30 day mortality		41%		49%		30.5%	
6 mth mortality						44.3%	
1 yr mortality			47%			44%	

* SAPS; **Apache 2; *** Apache 3

**Table 7:** ICU studies of mechanically ventilated patients receiving neuromuscular blockers

	**Newman**^52^	**Rudis**^53^	**Kupfer**^54^	**Douglass**^55^	**Prielipp**^4^	**Segredo**^56^
Study type	Prospective	Prospective	Prospective	Retrospective	Prospective	Prospective
Randomized	Yes	Yes	No	No	Yes	No
Patients	ICU	ICU	VEC > 6 hrs	Asthma	ICU	VEC>24 hrs
# of subjects	61	77	10	25	54	16
Mean age(yrs)	51	54	34	39	49	
Apache score	18	73			27	
Neuromuscular blocker	CISATRA (n = 40) ATRA (n = 21)	VEC (n = 35 standard Assessment; N= 42 nerve stim) 65 survivors	VEC	VEC 22 of 25 pts	CISTATRA (n = 28) VEC (n = 30)	VEC
Dose (mean)	CISATRA 3.1 ug/kg/min ATRA 10.4 ug/kg/min	VEC load 0.08 mg/kg infusion 0.08 mg/kg/hr dosing individualized		492 mg (SD692 mg)	CISTATRA 2.6 mg/kg/hr VEC 0.9 mg/kg/hr twitch monitor	
Duration of infusion	47 hrs	Standard assessment- 55.1 +/- 34.3 hrs Nerve stimulation 43.2 +/- 31.8		6.6 days	CISTATRA 80 +/- 7 h VEC 66 +/- 12 h.	
Recovery from block	1 hour 70% TOF for both drugs	50% of control pts recovery was 3.5 hrs (95% CI 2–8) vs. 1.7 hrs (95% CI 1–2) in nerve stim patients	7/10 pts with weakness, 3/10 muscle wasting, 2/10 difficulty weaning		70% TOF ratio CISTATRA 68 +/- 13 min VEC 387 +/- 163 min, longer (P = 0.02)	7 of 16 pts had prolonged block (l6 hrs – 7 days)
Neuro- muscular Outcome	No patient showed evidence of weakness following discontinuation of either CISATRA or ATRA	Median time for 50% of control pts to breathe spontaneously was 4.8 hrs (95% CI 3–9) compared with 2 hrs (95% CI 2–5) 11 of 35 control pts had prolonged block (>4 hr) 5/42 perip nerve stim pts had long block (p < .05) 3 survivors needed physical therapy for 35 to 137 days	Pts with polyneuropathy 1352 mg in 7.2 days Without polyneuropathy 528 mg for 3.8 days (p0.04)	9/25 had weakness Patients with myopathy had significantly higher total dose of VEC (p < 0.001)	Prolonged recovery CISTATRA: 2 patients VEC : 13 patients P = 0.002 1 VEC patient significant myopathy	
	**deLemos**^57^	**Khuenl-Brady**^58^	**Leatherman**^59^	**Coakley**^60^	**Murray**^61^	**Coakley**^62^
Study Type	Prospective	Prospective	Retrospective	Prospective	Prospective	Prospective
Randomized	No	No	No	No	Yes	No
Patients	ICU	Block > 2 days	Asthma	ICU >7 days	ICU	ICU >7 days
# of subjects	30	60	107	44	40	23
Mean age (yrs)	42	36		60	52	55
Apache	26			19	27	15.9
Neuromusc. blocker	PANC with TOF titrate	PANC (n = 30) PIPE (n = 30)	ATRA, PANC, VEC		DOX, PANC	15 of 23 received
Dose (mean)	Intermittent group (n = 14) 0.02 mg/kg/hr) Continuous Infusion (n = 16) .04 mg/kg/hr	3 mg/h with both			DOX (0.04 mg/kg) PANC (0.07 mg/kg)	
Duration Of infusion	6 days	> 48 hrs		> 7 days	2.5 days	
Recovery from block	Median time to recover from paralysis was 3.5 hrs (1.82–5.18) in infusion group vs. 6.3 hrs (3.40–9.19) in intermittent bolus group (p =.10)		Corticosteroids associated with more muscle weakness 20 of 69 versus 0 of 38 (p < 0.001)			
Neuro- muscular Outcome	5 in the infusion group and 1 intermittent had persistent severe muscle weakness 3 from each group had prolonged recovery >12 hrs.	None of the patients had muscle weakness	20 weak patients were paralysed longer 3.4 +/- 2.4 versus 0.6 +/- 0.7 d (p < 0.001) 18 of 20 weak pts paralysed > 24 h.	19 had motor & sensory findingss no relationship between neurophys. abnormality & APACHE II score, organ failure, sepsis, muscle relaxant, or steroids Mortality 23%	DOX shorter recovery time after >2 days of administration. (279.8 vs. 138.8 mins) no cases of prolonged neuromuscular block	10/23 had EMG, 9 of 10 had axonopathy, 8 were sensorimotor Mortality 21%

**Figure 2:**
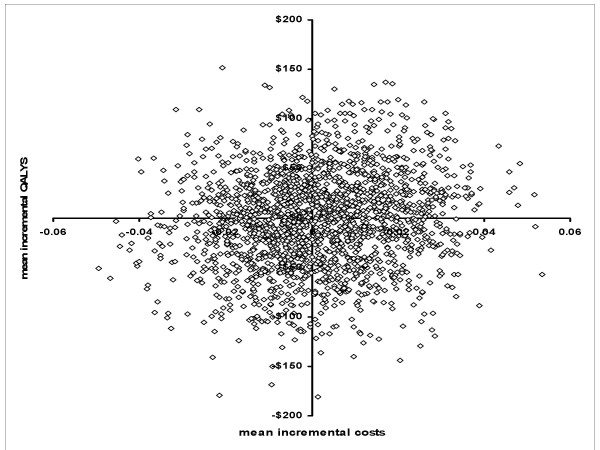
**The results of computer modeling**. The x- axis has the mean incremental QALYs and the y-axis has the mean incremental costs for the 10,000 Monte-Carlo simulations, each represented by a dot.

Assuming society would be willing to pay $35,000 for an additional quality adjusted life (i.e., ceiling ratio), even if a drug (that cost $267 more) did reduce AQMS from 25% to 21% and decrease intubation time by 6 hours, the net monetary benefit would only equal $137. By running repeated iterations of the model, Figure 2 has a scatter plot of the joint distribution of the mean incremental costs (mean decrease US $96, SD $5,134) and mean incremental QALYs (mean increase of 0.12, SD 0.0159) gained for bootstrap samples. For the base case, the net monetary benefit of reducing both AQMS and ventilation time would be positive for only 51% of patients.

### Sensitivity analysis

The net monetary benefit was positive for 50% of simulations with a ceiling ratio of $1,000 versus 51% if the ceiling ratio was increased to $100,000. The lack of sensitivity was caused by the mean changes in QALY and cost to be small relative to their standard deviations.

The variables that had the largest influence on the results, from most to least important, were probability from ICU intubated to death, probability from ICU intubated to extubated, and probability from ICU extubated to ward. The better the patients do overall, the larger the net monetary benefit of a drug that reduces AQMS and/or intubation times.

## Discussion

To properly allocate research money, computerized economic modeling should be first used to determine whether a pharmacoeconomic study can be expected to have a significant finding and thus be undertaken. ARDS patients receiving a neuromuscular blocker have a high mortality, and unpredictable outcome, which results in large variability in costs per case. If a patient dies, there is no benefit to any drug or intervention that reduces ventilation time or AQMS incidence. Consequently, a prospective, randomized pharmacoeconomic study of neuromuscular blockers in the ICU to assess AQMS or intubation times is impractical.

Published studies comparing neuromuscular blockers in the ICU for ARDS are limited by the heterogeneity of study methods and outcomes (e.g., each study defined myopathy/weakness differently). We found that the best way to increase net monetary benefit would be for the drug to affect the key variable – the chance of a patient dying. The benefit in spending extra money on such a drug is more likely to be important in a subset of critically ill patients identified as having prolonged intubation and a low chance of death.

### Neuromuscular blockers and recovery

The optimal balance between sedation and paralysis in ARDS patients is unclear. Since the probability of having a positive net monetary benefit is only for 51% of patients, practitioners choosing neuromuscular blockers need to consider risk factors for a patient developing AQMS such as female gender, the number of days with dysfunction of two or more organs, duration of mechanical ventilation, and administration of corticosteroids. It may be that the recognition of the problem of AQMS and consequent avoidance of or decrease in dosing of neuromuscular blockers, particularly when corticosteroids are given concurrently, in the ICU has reduced the incidence of AQMS [19].

### Assessing validity of computer modeling

The challenge is to design a useful model. Had we chosen to evaluate just the portion of care that occurs in the ICU, there would be less clinical uncertainty, because we would neglect what happened to the patient after ICU discharge. On the other hand, by including a six-month time frame, there is increasing uncertainty related to the complex course of the ARDS.

We tested the robustness of our modeling by comparing what our computer simulation predicted with published studies documenting the natural clinical progression of ARDS patients. For example, our model predicted that 28% of patients (base case being a 55 year- with pneumonia ARDS receiving neuromuscular blockade for 3.5 days) would be discharged home after 6 months, and that 18% would receive care in a long-term care facility. Both endpoints are consistent with a previous study of discharge disposition [20]. Our model also predicts that patients would have 15 ventilator free days, which matches well with published studies [21]. Thus, the scattergram obtained in Figure 2 reflects the uncertainties about how the probabilities will change if a new drug is used to reduce delays in neuromuscular recovery.

### Costing issues

Each facility may be able to negotiate individual contracts for neuromuscular blockers so the cost differences we estimated based on average wholesale price may not apply to a particular ICU.

Prolonged recovery from neuromuscular blockade may add costs due to additional sedative drugs, mechanical ventilation, and physician and nurse, ICU time. Importantly, the majority of the costs of treating patients with ARDS are spent on those who eventually die [22]. A detailed costing study of 193 critically ill adults found that factors such as severity of illness, gender, age, mechanical ventilation, emergency admission, and mortality were only able to explain 34% of the variation in average daily costs [23].

It may be that a good way to reduce time on mechanical ventilation is to have a full time intensivist rounding in the ICU 10–12 hours a day repeatedly evaluating the patient for extubation. However, in many ICUs this may not be available. A six-hour reduction in time on the ventilator may not be applicable in such settings. Although from society's perspective six-hours of ventilator time is important and measurable, from the hospital's perspective most of the cost of ICU care is a fixed cost. Even if a patient spends three hours less in the ICU, institutional costs may not be affected significantly because of the high overhead costs of hospital care. Different ICUs will have a different proportion of variable costs. In fact, nursing labor productivity is most sensitive to the number of admissions to the ICU each year and the method of compensating nurses (e.g., salary or hourly).

## Conclusion

The multifactorial etiology of AQMS, the highly variable clinical course of patients with ARDS as well as the disease's high mortality rate, makes the determination of whether selection of certain neuromuscular blockers decreases the incidence of AQMS or reduces intubation time difficult to answer. Our simulation computer model predicted the mean cost for ARDS patients receiving standard care for 6 months to be $62,238, with an overall 6-month mortality of 39%. Although it would be important to determine if a particular neuromuscular blocker diminishes the incidence of AQMS, a prospective, randomized pharmacoeconomic study of neuromuscular blockers in the ICU is impractical because of the highly variable clinical course of patients with ARDS.

## Competing interests

This study was funded in part by Abbott Laboratories, 100 Abbott Park Road Abbott Park, Illinois. Abbott Laboratories did not participate in the collection, analysis, or interpretation of the results contained within the manuscript.

## Authors' contributions

AM conceived the study, outlined the economic model, and wrote the manuscript. JLC led the literature review, and provided expert clinical guidance as to the clinical message. FD participated in the design of the study and performed the statistical analysis. All authors read and approved the final manuscript.

## Pre-publication history

The pre-publication history for this paper can be accessed here:


